# A Biomimetic Gazelle Optimization Approach for Enhanced Temperature Regulation in Electric Furnaces

**DOI:** 10.3390/biomimetics11040255

**Published:** 2026-04-07

**Authors:** Davut Izci, Adil Ozcayci, Serdar Ekinci, Irfan Okten, Erdal Akin, Gokhan Yuksek, Ali Akdagli, Ali Yildiz, Filiz Karaomerlioglu

**Affiliations:** 1 Department of Electrical and Electronic Engineering, Bursa Uludag University, 16059 Bursa, Turkey; davutizci@uludag.edu.tr; 2 Department of Computer Engineering, Bitlis Eren University, 13100 Bitlis, Turkey; sekinci@beu.edu.tr (S.E.); iokten@beu.edu.tr (I.O.); 3 Department of Computer Science and Media Technology, Malmö University, 205 06 Malmö, Sweden; 4 Sustainable Digitalisation Research Centre, Malmö University, 205 06 Malmö, Sweden; 5 Biofilms Research Center for Biointerfaces (BRCB), Malmö University, 205 06 Malmö, Sweden; 6 Department of Electrical and Electronics Engineering, Batman University, 72100 Batman, Turkey; gokhan.yuksek@batman.edu.tr; 7 Department of Electrical and Electronics Engineering, Mersin University, 33343 Mersin, Turkey; akdagli@mersin.edu.tr (A.A.); yildiz@mersin.edu.tr (A.Y.); filizkrm@mersin.edu.tr (F.K.)

**Keywords:** nature-inspired metaheuristics, gazelle optimization algorithm, PID controller, electric furnace temperature system, stability

## Abstract

Accurate temperature regulation is essential for ensuring product quality, operational safety, and energy efficiency in industrial electric furnace systems. However, the inherent thermal inertia, time-delay effects, and nonlinear dynamics of furnace processes often make precise temperature control a challenging task. Motivated by these challenges, this study proposes an optimization-based control framework aimed at improving the temperature regulation performance of electric furnace systems. The proposed approach integrates a proportional–integral–derivative (PID) controller with the recently developed gazelle optimization algorithm (GOA) for automatic tuning of the controller parameters. First, a mathematical model of the electric furnace is established to describe the dynamic relationship between the control input and the furnace temperature output. Based on this model, a PID controller is implemented to regulate the furnace temperature. The parameters of the PID controller are then optimized using GOA, a nature-inspired metaheuristic algorithm that mimics the adaptive predator–prey survival strategies observed in gazelle herds. In order to achieve a balanced improvement in both steady-state and transient performance, a composite objective function is introduced. The proposed performance index combines the integral of absolute error with additional transient performance indicators related to maximum overshoot and settling time. The effectiveness of the proposed GOA-based tuning framework is evaluated through extensive simulation studies and statistical analyses conducted over multiple independent optimization runs. The results demonstrate stable convergence behavior, with the optimization process achieving a minimum objective value of 2.4251, a maximum value of 2.5347, and an average value of 2.4674 across 25 runs. The optimized control system exhibits improved dynamic characteristics, including a rise time of 1.8509 s, a settling time of 3.6834 s, and a low overshoot of 1.5104%. To further assess its effectiveness, the proposed GOA–PID control strategy is compared with several widely used controller tuning methods reported in the literature, including genetic algorithm, Ziegler–Nichols, Cohen–Coon, Nelder–Mead, and direct synthesis approaches. Comparative results indicate that the proposed method achieves a superior balance between response speed, stability, and temperature tracking accuracy.

## 1. Introduction

Electric furnaces constitute a fundamental component of modern industrial production systems, particularly in processes that require controlled high-temperature environments. These systems are widely employed in the production and processing of metals, alloys, ceramics, glass, and numerous chemical products, where precise thermal conditions must be maintained to ensure proper material transformation and product quality [1,2,3,4]. In industries such as metallurgy and advanced materials manufacturing, electric furnaces enable controlled heating, melting, and refining operations that are essential for converting raw materials into usable industrial products [5]. Owing to their ability to provide stable and controllable temperature environments, electric furnaces have become indispensable tools in contemporary manufacturing infrastructures.

In many industrial applications, the quality and efficiency of production processes are strongly influenced by the accuracy of temperature regulation. Electric furnaces are typically required to operate within carefully defined temperature ranges in order to ensure the desired metallurgical or chemical properties of the processed materials. Excessive temperature deviations may result in structural defects, undesired chemical reactions, or degradation of the final product, whereas insufficient heating may prevent the required material transformations from occurring. Consequently, reliable temperature control plays a central role in maintaining product quality, minimizing thermal damage, and improving energy utilization within furnace-based industrial systems [6,7,8].

From an operational perspective, the performance of an electric furnace is directly governed by its control system. Inefficient temperature regulation may lead to excessive energy consumption, prolonged processing times, and unstable thermal behavior. In addition, poorly controlled heating processes may introduce thermal stresses within furnace components, thereby shortening equipment lifespan and increasing maintenance requirements. Such inefficiencies may also contribute to elevated operational costs and increased environmental impact. For these reasons, the development of effective and robust control strategies for electric furnace temperature regulation has become a significant research topic in industrial control engineering [9].

Over recent decades, a wide variety of control strategies and optimization techniques have been investigated to enhance the performance of temperature control systems in electric furnaces. Classical control approaches, intelligent control techniques, and optimization-based tuning methods have all been explored with the aim of improving response speed, stability, and energy efficiency. For instance, Rawat et al. [10] conducted a comparative investigation of linear quadratic regulator (LQR) and proportional–integral–derivative (PID) control strategies for electric furnace temperature systems, highlighting the advantages and limitations of each method under different operating conditions.

In addition to classical approaches, intelligent control techniques have also been widely explored. Ghanim and Ajel [11] proposed an optimal fuzzy logic control framework in which controller parameters were tuned using the social spider optimization (SSO) algorithm. Their results demonstrated that combining fuzzy logic with metaheuristic optimization could improve temperature regulation accuracy compared with conventional control methods. Similarly, Pringsakul and Puangdownreong [12] introduced a PID acceleration (PIDA) controller whose parameters were optimized using the modified flower pollination algorithm (MoFPA), leading to improvements in transient response characteristics, particularly in terms of reduced overshoot and faster settling behavior.

More recently, advanced control strategies have been developed to address the nonlinearities, uncertainties, and disturbances inherent in industrial thermal systems. Hussein et al. [13] proposed a modern temperature control approach based on a modified optimization technique to enhance system stability and response speed. Rsetam et al. [14] investigated a robust adaptive active disturbance rejection control (ADRC) scheme combined with a continuous sliding mode component, enabling satisfactory performance under uncertain operating conditions and external disturbances. Optimization-based fuzzy control strategies have also been reported. Ajorloo et al. [9] developed a mathematical model for a vacuum box electric furnace and designed an optimized fuzzy temperature controller, demonstrating stable and accurate regulation through both simulation and experimental validation. In another study, Moussa [15] proposed an adaptive lag compensator tuned using the improved gorilla troops optimization (IGTO) algorithm, emphasizing enhanced energy efficiency while maintaining satisfactory control performance. Furthermore, Liu et al. [16] introduced a fuzzy fractional-order PID controller for industrial temperature processes, showing that hybrid intelligent control frameworks can improve both robustness and control accuracy in complex environments.

The studies discussed above clearly indicate a growing interest in integrating optimization algorithms and intelligent control techniques for improving electric furnace temperature regulation. Although these approaches have demonstrated promising results, the search for efficient and reliable controller tuning strategies remains an active research area. In particular, metaheuristic optimization algorithms have attracted considerable attention due to their ability to explore complex search spaces effectively and identify high-quality solutions without requiring gradient information.

Motivated by these developments, this study proposes a temperature control framework in which the parameters of a PID controller are optimized using the gazelle optimization algorithm (GOA) [17]. GOA is a recently introduced nature-inspired metaheuristic that models the adaptive survival behaviors of gazelles in predator–prey environments. The algorithm incorporates mechanisms that mimic grazing movements, escape strategies, and predator pursuit dynamics to achieve a balance between exploration and exploitation during the search process.

Among the many metaheuristic optimization algorithms proposed in recent years, GOA has attracted attention due to its ability to maintain an effective balance between global exploration and local exploitation. Its adaptive movement strategies enable diversified search behavior in the early stages of optimization while progressively concentrating on promising regions of the search space. The effectiveness of GOA has been demonstrated in several engineering applications. For example, it has been successfully applied to system identification problems, prediction of machining characteristics in carbon fiber–reinforced materials, path planning, and speed control of electric motors [18,19,20,21,22,23]. Furthermore, GOA has been utilized for parameter estimation of lithium-ion batteries in smart grid applications [24] and for optimization of microgrid operation strategies [25]. These studies highlight the versatility and effectiveness of GOA as a modern optimization tool for solving complex engineering problems.

The characteristic of GOA is particularly advantageous for PID tuning problems, where the optimization landscape is nonlinear and may contain multiple local optima. Accordingly, GOA is employed in this study as a flexible and efficient optimization tool for determining suitable PID controller parameters for electric furnace temperature regulation. The PID controller is adopted in this study due to its widespread use in industrial systems and its well-established reliability. Despite the availability of more advanced control strategies, PID controllers remain popular because of their simple structure, ease of implementation, and satisfactory performance in many practical applications. Successful PID-based control solutions have been reported in a wide range of systems, including robotic manipulators [26], pulp neutralization processes [27], DC motor speed regulation [28], load frequency control in microgrids [29], and liquid level control systems [30]. PID controllers have also been effectively applied in automatic voltage regulation and doubly fed induction-generator-based wind turbine systems [31].

In addition to employing GOA for controller tuning, this study introduces a modified objective function for evaluating controller performance. The proposed performance criterion is derived from a modified integral of absolute error (*IAE*) metric [32]. By incorporating additional performance indicators into the objective function, the formulation aims to reduce excessive overshoot and long settling times while maintaining accurate temperature tracking.

The effectiveness of the proposed GOA-based PID tuning strategy is evaluated through extensive simulation studies. Statistical analysis indicates that the optimization process consistently identifies high-quality controller parameters across multiple independent runs. Specifically, the proposed approach achieves a minimum objective function value of 2.4251, a maximum value of 2.5347, and an average value of 2.4674. The relative variation of approximately 4.4425% demonstrates stable and consistent performance across different runs. Furthermore, the convergence characteristics reveal a steady reduction in the objective function value over successive iterations, indicating that the optimization process effectively guides the search toward improved solutions. The resulting step response characteristics confirm that the optimized control system achieves a rapid rise time, short settling time, and minimal overshoot.

To further assess the effectiveness of the proposed method, the GOA-based PID controller is compared with several established tuning approaches reported in the literature, including the genetic algorithm [33], Ziegler–Nichols method [34], Cohen–Coon method [34], Nelder–Mead optimization [34], and the direct synthesis technique [34]. The comparative analysis demonstrates that the proposed approach provides improved transient response characteristics and more stable temperature regulation compared with these widely used methods. Overall, the results indicate that integrating GOA with a PID controller offers an effective and reliable solution for electric furnace temperature regulation, contributing to improved system stability, faster transient response, and enhanced control accuracy in practical industrial applications.

The remainder of this paper is organized as follows. Section 2 presents the methodology of the study. In this section, the GOA is first described, after which the optimization problem is formulated through the electric furnace temperature control model, the PID controller structure, and the proposed GOA-based tuning framework. Section 3 is devoted to simulation and analysis. In this section, the statistical performance of the optimizer, the evolution characteristics of the objective function and controller parameters, the step response of the GOA-PID controlled system, and the comparative evaluation with reported tuning methods are presented and discussed in detail. Finally, the main findings, limitations, and potential future research directions are summarized in Section 4.

## 2. Methodology

### 2.1. Gazelle Optimization Algorithm

The gazelle optimization algorithm (GOA) is a nature-inspired metaheuristic that emulates the collective survival strategies observed in gazelle herds [17]. In natural ecosystems, gazelles must continuously balance two conflicting objectives: searching for food while simultaneously maintaining awareness of potential predators. Their survival depends on adaptive movement patterns that combine cautious local exploration during grazing and sudden large displacements when escaping threats. These behavioral patterns are mathematically translated into an optimization framework capable of effectively exploring complex search spaces.

In GOA, each gazelle is modeled as a candidate solution to the optimization problem. A population of n gazelles is maintained, where each gazelle is described by a position vector in a d-dimensional search space. The entire population can therefore be represented by the matrix $X\in R^{n\times d}$.

(1) $$X=\left[\begin{array}{cccc} x_{1,1} & x_{1,2} & \cdots & x_{1,d}\\ x_{2,1} & x_{2,2} & \cdots & x_{2,d}\\ \vdots & \vdots & \ddots & \vdots \\ x_{n,1} & x_{n,2} & \cdots & x_{n,d} \end{array}\right]$$

In this representation, $x_{i,j}$ denotes the value of the $j^{th}$ decision variable associated with the $i^{th}$ gazelle. The dimension d corresponds to the number of variables of the optimization problem, while n denotes the population size. At the beginning of the search process, gazelle positions are distributed randomly within the feasible search domain. For each dimension j, the allowable range is specified by a lower bound $LB_{j}$ and an upper bound $UB_{j}$. The initial position of each gazelle is therefore generated according to $x_{i,j}=LB_{j}+rand\times (UB_{j}-LB_{j})$ where rand is a uniformly distributed random number within the interval [0,1]. This initialization strategy ensures that the initial population is dispersed throughout the search space, promoting global exploration. During the optimization process, some gazelles exhibit superior performance in terms of objective function value. These individuals act as leaders that guide the movement of the rest of the population. The positions of such promising solutions are stored in an elite matrix, given in Equation (2):

(2) $$Elite=\left[\begin{array}{cccc} x_{1,1}^{\prime } & x_{1,2}^{\prime } & \cdots & x_{1,d}^{\prime }\\ x_{2,1}^{\prime } & x_{2,2}^{\prime } & \cdots & x_{2,d}^{\prime }\\ \vdots & \vdots & \ddots & \vdots \\ x_{n,1}^{\prime } & x_{n,2}^{\prime } & \cdots & x_{n,d}^{\prime } \end{array}\right]$$

where $x_{i,j}^{\prime }$ denotes the $j^{th}$ coordinate of a leading gazelle. The elite set is continuously updated whenever a more favorable solution is discovered. When gazelles are grazing in a safe environment, their movement is typically gradual and localized. Such behavior is modeled using stochastic perturbations generated from Brownian motion. Brownian motion follows a Gaussian probability distribution with mean μ and variance $\sigma^{2}$. Its probability density function can be expressed as follows.

(3) $$f_{B}(x,\mu ,\sigma )=\frac{1}{\sqrt{2\pi \sigma^{2}}}exp\left(-\frac{\left(x-\mu {)}^{2}\right.}{2\sigma^{2}}\right)$$

In most implementations of GOA, the parameters are selected as μ=0 and $\sigma^{2}=1$, which produces small random steps around the current position. Based on this stochastic model, the grazing movement of a gazelle can be expressed as:

(4) $$g_{i+1}=g_{i}+s\cdot R\cdot R_{B}\cdot (Elite_{i}-R_{B}\cdot g_{i})$$

where $g_{i}$ denotes the current position of a gazelle and $g_{i+1}$ represents the updated position in the next iteration. The parameter s controls the grazing speed, while R is a vector of uniformly distributed random numbers in [0,1]. The vector $R_{B}$ represents random variables generated according to Brownian motion. In contrast to grazing behavior, the presence of predators triggers abrupt escape maneuvers. Gazelles often perform irregular long jumps to evade threats, which can be mathematically described using Lévy flight. Lévy flight is a stochastic process characterized by many small steps interspersed with occasional long-distance movements. The probability distribution of the step length $x_{j}$ can be approximated by $L(x_{j})\propto {|x}_{j}{|}^{1-\alpha }$, where α is the Lévy stability parameter, typically selected within the interval 1<α<2. A more general representation of the Lévy stable distribution is given by:

(5) $$f_{L}(x;\alpha ,\gamma )=\frac{1}{\pi }\int_{0}^{\infty }exp(-\gamma q^{\alpha })cos(qx)\;dq$$

where γ is a scaling factor controlling the spread of the distribution. In practical implementations, Lévy steps are generated using:

(6) $$Levy(\alpha )=0.05\times \frac{x}{|y{|}^{1/\alpha }}$$

where x and y are random numbers drawn from normal distributions with zero mean. The variances of these distributions are denoted by $\sigma_{x}^{2}$ and $\sigma_{y}^{2}$, respectively, while α is commonly set to 1.5. The parameter $\sigma_{x}$ can be calculated as:

(7) $$\sigma_{x}={\left(\frac{\Gamma (1+\alpha )sin(\pi \alpha /2)}{\Gamma ((1+\alpha )/2)\alpha 2^{(\alpha -1)/2}}\right)}^{1/\alpha }$$

where Γ(⋅) represents the Gamma function. When predator detection occurs, gazelles adjust their motion according to the Lévy flight escape strategy. The position update mechanism can be written as:

(8) $${\vec{g}}_{i+1}={\vec{g}}_{i}+S\cdot \mu \cdot \vec{R}\cdot \vec{R_{L}}\left({\vec{Elite}}_{i}-\vec{R_{L}}{\vec{g}}_{i}\right)$$

where S denotes the maximum running speed of the gazelle and $\vec{R_{L}}$ is a random vector generated according to the Lévy distribution. Meanwhile, the chasing behavior of the predator is modeled through a Brownian-motion-based update rule given below.

(9) $${\vec{g}}_{i+1}={\vec{g}}_{i}+S\cdot \mu \cdot CF\cdot \vec{R_{B}}\left({\vec{Elite}}_{i}-\vec{R_{L}}{\vec{g}}_{i}\right)$$

The parameter CF represents the cumulative effect of predator pressure and gradually decreases during the search process. It is calculated as:

(10) $$CF={\left(1-\frac{iter}{iter_{max}}\right)}^{2\frac{iter}{iter_{max}}}$$

where iter and $iter_{max}$ denote the current iteration number and the maximum number of iterations, respectively.

To further maintain diversity within the population and reduce the likelihood of premature convergence, a predator success rate (PSR) mechanism is incorporated. This mechanism probabilistically determines whether a gazelle escapes toward a random region of the search space or moves relative to other members of the herd. The corresponding update rule can be expressed as:

(11) $${\vec{g}}_{i+1}=\left\{\begin{array}{cc} {\vec{g}}_{i}+CF[(\vec{LB}+\vec{R}(\vec{UB}-\vec{LB}))\vec{U}], & r\leq PSRs\\ {\vec{g}}_{i}+[PSRs(1-r)+r]({\vec{g}}_{r1}-{\vec{g}}_{r2}), & r>PSRs \end{array}\right.$$

where r is a random number in the interval [0,1], PSRs represents the predator success rate parameter, and $\vec{U}$ is a binary vector generated from a random variable U. Specifically, $\vec{U}=0$ when U<0.34 and $\vec{U}=1$ otherwise. The indices $r_{1}$ and $r_{2}$ correspond to two randomly selected gazelles from the population. Through the coordinated interaction of grazing movement, predator–prey dynamics, and Lévy-based escape strategies, GOA achieves a balance between global exploration and local exploitation. This balance enables the algorithm to efficiently search complex optimization landscapes and identify high-quality candidate solutions.

**Figure 1:**
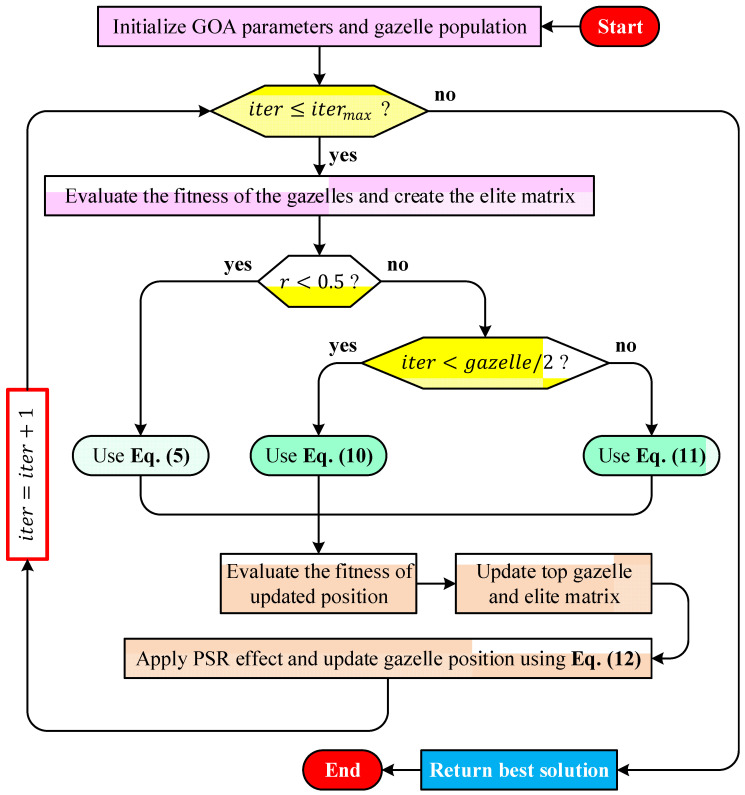
Flowchart of the proposed GOA optimizer.

The overall workflow of the algorithm is illustrated in the flowchart presented in Figure 1. The procedure begins with the initialization of the algorithmic parameters and the generation of the initial gazelle population within the predefined search boundaries. Subsequently, the objective function values of all gazelles are evaluated, and the most promising individuals are identified to construct the elite matrix that guides the population during the search process. At each iteration, a stochastic decision mechanism determines the movement strategy to be applied. A random variable r is generated to select between alternative behavioral patterns that mimic grazing, predator avoidance, or pursuit dynamics. Depending on this condition and the current stage of the search, the gazelle positions are updated using one of the position update mechanisms described previously in Equations (4), (8), or (9). These update rules represent different movement patterns that collectively balance exploration of the search space and exploitation of promising regions. After the positions of the gazelles are updated, the fitness of the new candidate solutions is recalculated. If an improved solution is discovered, the elite matrix and the position of the best gazelle are correspondingly updated. To further enhance diversity and reduce the risk of stagnation in local optima, the PSR mechanism is subsequently applied, and gazelle positions are refined using the update rule given in Equation (11). The iteration counter is then incremented, and the procedure is repeated until the predefined maximum number of iterations is reached. Upon termination of the iterative process, the best solution obtained throughout the search is returned as the final outcome of the algorithm.

### 2.2. Definition of Optimization Problem

#### 2.2.1. Temperature Control System

**Figure 2:**
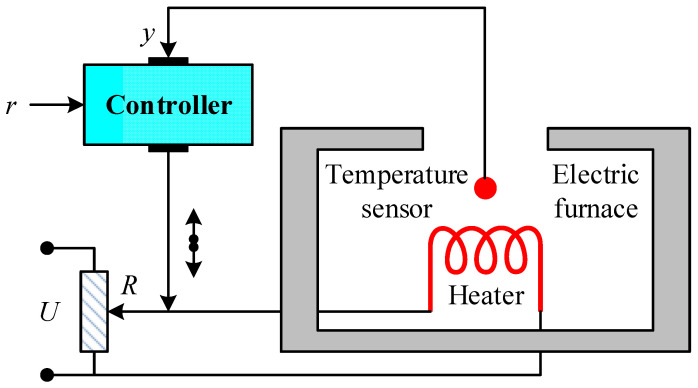
Block diagram representing the temperature control system adopted for this study.

An electric furnace temperature regulation system typically consists of three fundamental components: the heating unit, a temperature sensing element, and a feedback controller [1]. In such systems, the objective is to maintain the furnace temperature at a desired reference level despite thermal inertia and process delays. The configuration adopted in this study is illustrated in Figure 2. Within this structure, the controller continuously processes the difference between the desired temperature and the measured temperature and generates a control signal that regulates the electrical power delivered to the heating element. As shown in Figure 2, the reference signal r represents the desired temperature setpoint that the system aims to achieve. The controller processes this reference together with the feedback signal obtained from the temperature sensor. The controller output signal is denoted by U, which corresponds to the voltage applied to the heating element through the actuator circuitry. The heater inside the furnace converts this electrical signal into thermal energy, causing the furnace temperature to change. The temperature sensor (thermocouple) measures the resulting temperature and produces a feedback signal y, which represents the measured output temperature. This feedback signal is returned to the controller to close the control loop. In the diagram, R denotes the electrical resistance associated with the actuator path that influences the power delivered to the heating element.

In order to analyze the dynamic behavior of the furnace, a mathematical model describing the relationship between the input signal and the output temperature is required. The thermal dynamics of the furnace can be approximated by a second-order system with a transport delay. The transfer function of the plant, denoted by $G_{p}(s)$, is expressed as [35]:

(12) $$G_{p}\left(s\right)=\frac{0.15}{s^{2}+1.1s+0.2}e^{-1.5s}$$

where the exponential term $e^{-1.5s}$ corresponds to a transport delay of 1.5 s, which arises from the thermal inertia and heat propagation within the furnace chamber. The presence of the time-delay term complicates analytical analysis and controller design. Therefore, a first-order Padé approximation [36] is employed to represent the delay term with a rational transfer function. This approximation allows the system to be analyzed using standard control techniques. After applying the first-order Padé approximation, the plant model can be expressed as follows.

(13) $$G_{p}\left(s\right)=\frac{-0.1125s+0.15}{0.75s^{3}+1.825s^{2}+1.25s+0.2}$$

This rational representation provides a convenient model for simulation and controller synthesis while preserving the essential dynamic characteristics of the original delayed system.

**Figure 3:**
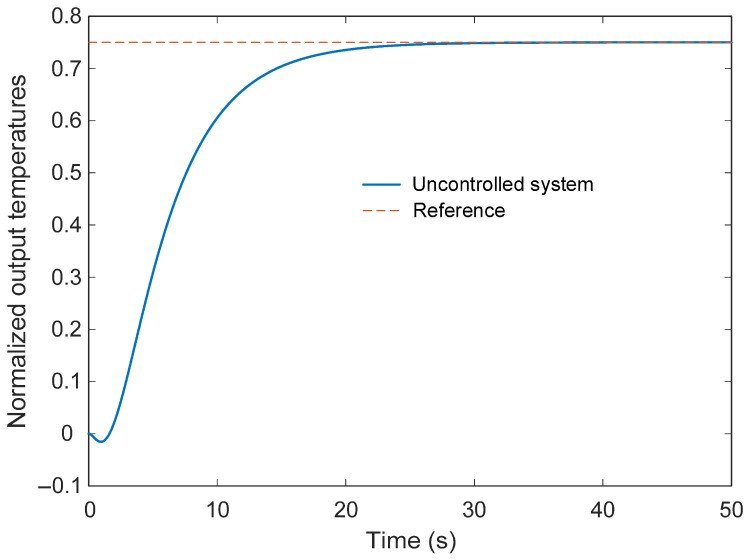
Step response of uncontrolled electric furnace temperature system.

To better understand the intrinsic behavior of the furnace dynamics, the open-loop step response of the plant without a controller is examined. The resulting response is presented in Figure 3. The response curve in Figure 3 reveals the inherent limitations of the uncontrolled system. When a unit step input is applied, the output temperature gradually approaches the reference level but exhibits slow dynamic behavior due to the thermal inertia of the process. The rise time of the system is approximately 10.22 s, indicating that the furnace temperature increases relatively slowly after the input change. The settling time is around 19.87 s, which reflects the time required for the temperature to stabilize near its steady-state value. It can also be observed that the response does not exhibit overshoot, which is typical for many thermal processes characterized by significant damping. However, the steady-state output reaches only about 0.75 of the normalized reference value, resulting in a steady-state error of approximately 0.25. This indicates that the open-loop furnace system is unable to track the desired temperature accurately without additional control action. These observations highlight the necessity of an effective feedback controller. Without a properly designed control strategy, the furnace exhibits slow transient dynamics and a persistent steady-state error, both of which can negatively affect temperature regulation performance in practical applications. Consequently, an appropriate control methodology must be developed to improve the response speed and eliminate the steady-state error while maintaining stable operation.

#### 2.2.2. PID Controller

Among classical control strategies, the PID controller remains one of the most widely adopted approaches in industrial process control due to its relatively simple structure and reliable performance in a wide range of applications [37]. In thermal systems such as electric furnaces, PID control is frequently employed to regulate temperature by continuously adjusting the heating power in response to deviations from the desired operating point. Through the combined action of proportional, integral, and derivative components, the controller modifies the control signal so that the furnace temperature tracks the prescribed reference value. The operation of the PID controller is based on the regulation of the error signal, which represents the difference between the desired temperature and the measured temperature of the furnace. Let r(t) denote the reference temperature (setpoint) and y(t) represent the measured furnace temperature obtained from the thermocouple. The error signal is therefore defined as e(t)=r(t)−y(t). The controller processes this error signal and generates the control input applied to the heating system. In the Laplace domain, the transfer function of a standard PID controller can be expressed as [38]:

(14) $$C\left(s\right)=K_{p}+\frac{K_{i}}{s}+K_{d}s$$

where C(s) denotes the controller transfer function. The parameters $K_{p}$, $K_{i}$, and $K_{d}$ represent the proportional gain, integral gain, and derivative gain, respectively. These parameters determine the influence of each control action on the overall system response. The proportional component $K_{p}$ produces a control effort that is directly proportional to the instantaneous error value. In the context of electric furnace temperature regulation, this term reacts immediately to deviations between the desired and measured temperature and adjusts the heating power accordingly. Increasing the proportional gain generally accelerates the response of the system; however, excessively large values may lead to oscillatory behavior. The integral component $K_{i}$ accounts for the accumulation of past errors over time. This action gradually increases the control effort whenever a persistent error exists. As a result, the integral term plays an important role in eliminating steady-state error, which is particularly relevant for thermal processes where slow dynamics may otherwise cause the system to settle at a temperature different from the desired setpoint. The derivative component $K_{d}$ is associated with the rate of change in the error signal. By predicting the future trend of the error based on its slope, the derivative action introduces a damping effect that improves transient stability and reduces overshoot. In thermal control applications, this predictive behavior helps prevent excessive heating and contributes to smoother temperature regulation.

**Figure 4:**
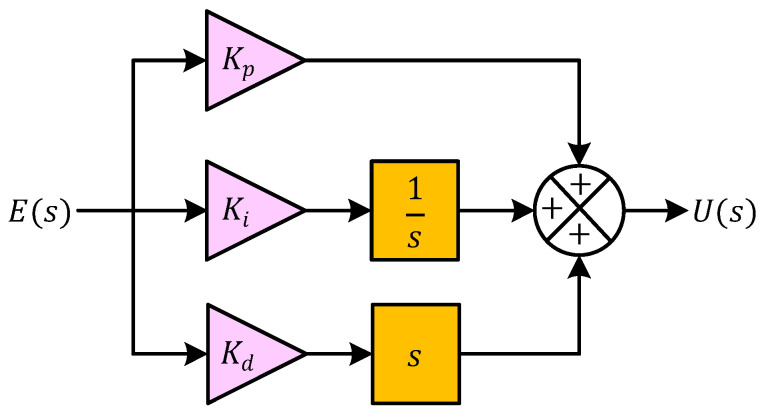
Block diagram demonstrating the contribution of each parameter of the PID controller.

The structural organization of the PID controller is illustrated in Figure 4. As depicted in Figure 4, the error signal E(s) is distributed into three parallel paths corresponding to the proportional, integral, and derivative components. In the proportional branch, the error signal is multiplied by the gain $K_{p}$. In the integral branch, the error is processed through the integrator 1/s and scaled by the gain $K_{i}$, thereby representing the accumulated error over time. In the derivative branch, the error signal is passed through the differential operator s and weighted by the gain $K_{d}$, which captures the rate of variation in the error. The outputs of these three branches are then combined at a summation node to produce the overall control signal U(s). Within the electric furnace temperature control system described previously, this control signal corresponds to the actuator input that regulates the electrical power delivered to the heating element. By adjusting the heater power based on the instantaneous, accumulated, and predicted error values, the PID controller enables the furnace temperature to follow the desired setpoint more accurately.

In practical applications, the performance of a PID controller depends heavily on the appropriate selection of its parameters $K_{p}$, $K_{i}$, and $K_{d}$. Improper tuning may lead to slow responses, oscillations, or excessive overshoot. For this reason, several tuning approaches have been proposed in the literature, including classical empirical techniques such as the Ziegler–Nichols and Cohen–Coon methods as well as modern optimization-based strategies. In the present work, the GOA [17] is employed to determine suitable PID parameters for the electric furnace temperature control system. By automatically searching for parameter combinations that improve the dynamic response, the optimization-based tuning process aims to achieve faster temperature tracking, reduced steady-state error, and improved overall stability of the furnace control system.

#### 2.2.3. Proposed Novel Approach for PID Controlled Temperature Control System

In many conventional PID-based control studies, controller performance is commonly assessed using error-based objective functions. One of the most frequently adopted criteria is the integral of absolute error (*IAE*) [39], which measures the accumulated magnitude of the control error over a specified time interval. The *IAE* performance index employed in this study is defined as:

(15) $$IAE=\int_{0}^{50}|e(t)|\times dt$$

where e(t) denotes the instantaneous control error, defined as the difference between the reference temperature (setpoint) and the measured furnace temperature at time t. The *IAE* metric evaluates the overall deviation of the system output from the desired trajectory during the observation period. Lower *IAE* values indicate improved tracking performance and smaller cumulative error.

Although the *IAE* criterion effectively penalizes large deviations from the setpoint, relying solely on this measure may not always lead to desirable transient behavior. In particular, minimizing only the error magnitude can result in responses with relatively large overshoot or longer settling times, which may be undesirable for thermal processes. In electric furnace systems, excessive overshoot may lead to overheating and energy inefficiency, while slow settling behavior can reduce process productivity and temperature stability. To address these limitations, a composite objective function is introduced in this work. The proposed formulation integrates the traditional *IAE* measure with additional transient performance indices in order to achieve a more balanced controller design. The objective function is defined as follows.

(16) $$W={(1-\theta }_{1}-\theta_{2})\times IAE+\theta_{1}\times M_{p}+\theta_{2}\times T_{s}$$

In this expression, the variable $M_{p}$ denotes the maximum overshoot, which represents the maximum percentage by which the system output exceeds the reference value during the transient response. The parameter $T_{s}$ represents the settling time, defined as the time required for the system response to remain within a tolerance band of ±2% around the desired steady-state value. The weighting coefficients $\theta_{1}$ and $\theta_{2}$ are introduced to regulate the relative importance of the different performance criteria. In this study, the parameters are selected as $\theta_{1}=0.15$ and $\theta_{2}=0.10$. These values were determined through preliminary experimentation in order to achieve a balanced compromise between minimizing the cumulative tracking error, limiting overshoot, and improving the settling speed of the system response. Consequently, the proposed objective function encourages a control solution that simultaneously improves steady-state accuracy and transient performance.

**Figure 5:**
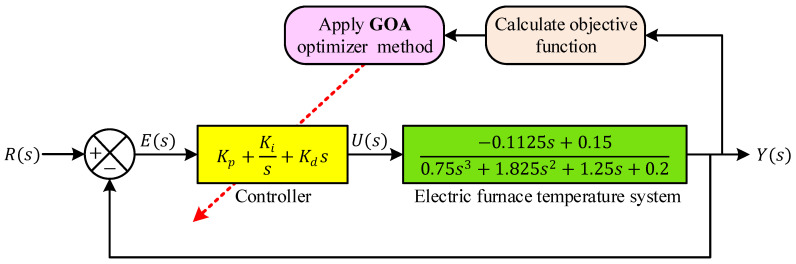
Structure of PID controlled system tuned by GOA.

The overall configuration of the proposed control framework is illustrated in Figure 5. As depicted in Figure 5, the reference signal R(s) represents the desired furnace temperature. This signal is compared with the measured output temperature Y(s) to produce the error signal E(s). The error is then processed by the PID controller. The controller generates the control signal U(s), which regulates the electrical power supplied to the heating element of the furnace. Within the proposed framework, the parameters of the PID controller ($K_{p}$, $K_{i}$, and $K_{d}$) are not selected manually. Instead, they are automatically determined using the GOA. During the optimization process, GOA generates candidate sets of PID parameters and evaluates their performance by simulating the closed-loop system response. The objective function defined in Equation (16) is then calculated for each candidate solution. Based on this evaluation, the optimizer iteratively updates the controller parameters in order to minimize the performance index W. Through this optimization-based tuning process, the controller parameters are adjusted such that the temperature of the electric furnace follows the reference signal with improved accuracy, reduced overshoot, and faster stabilization. As a result, the proposed approach enhances both the transient and steady-state characteristics of the temperature control system, making it more suitable for practical thermal process applications where precise and reliable temperature regulation is required.

## 3. Simulation and Analysis

In order to evaluate the effectiveness of the proposed optimization-based control framework, a series of simulation studies were conducted on the electric furnace temperature control system. The simulations were designed to assess the capability of the GOA to determine suitable PID controller parameters that improve the transient and steady-state performance of the closed-loop system. The principal algorithmic settings used during the optimization process are summarized in Table 1. As indicated in Table 1, the optimization procedure was carried out using a population size of 30 candidate solutions. Each candidate solution represents a potential set of PID controller parameters, which are iteratively updated by the GOA during the search process. The maximum number of iterations was set to 50, allowing the algorithm to sufficiently explore the search space while maintaining a reasonable computational effort. Since three controller parameters—namely the proportional, integral, and derivative gains—are optimized simultaneously, the number of decision variables in the optimization problem was defined as three. To evaluate the stability and repeatability of the optimization results, the GOA was executed 25 independent times under identical conditions. This repeated execution allows the influence of the stochastic nature of the algorithm to be examined and provides a reliable basis for statistical analysis of the obtained solutions.

Appropriate search boundaries were defined for the PID controller parameters in order to ensure that the optimization process remains within physically meaningful and stable regions of the parameter space. The proportional gain $K_{p}$ was allowed to vary within the interval [1, 4], while the integral gain $K_{i}$ was restricted to the range [0, 2]. Similarly, the derivative gain $K_{d}$ was constrained between [3, 7]. These limits were selected based on preliminary experimentation and general guidelines for thermal control systems, ensuring that the controller remains capable of achieving both adequate responsiveness and stable system behavior. In addition to the PID parameter limits, several internal parameters associated with the GOA were specified to regulate the search dynamics of the algorithm. The parameter s, which influences the stochastic movement behavior of the gazelle agents, was defined within the interval [0, 1], while the parameter μ was allowed to vary within [−1, 1]. Furthermore, two algorithm-specific constants were adopted to maintain a balanced search process. The parameter S was set to 0.88, and the predator success rate parameter PSRs was selected as 0.34. These values help regulate the interaction between exploration and exploitation phases during the optimization process. By adopting the parameter settings summarized in Table 1, the GOA was configured to perform an efficient search within the defined solution space for the PID controller gains. The following subsections present a detailed analysis of the optimization performance and the resulting control characteristics of the electric furnace temperature regulation system.

### 3.1. Statistical Success of GOA

**Figure 6:**
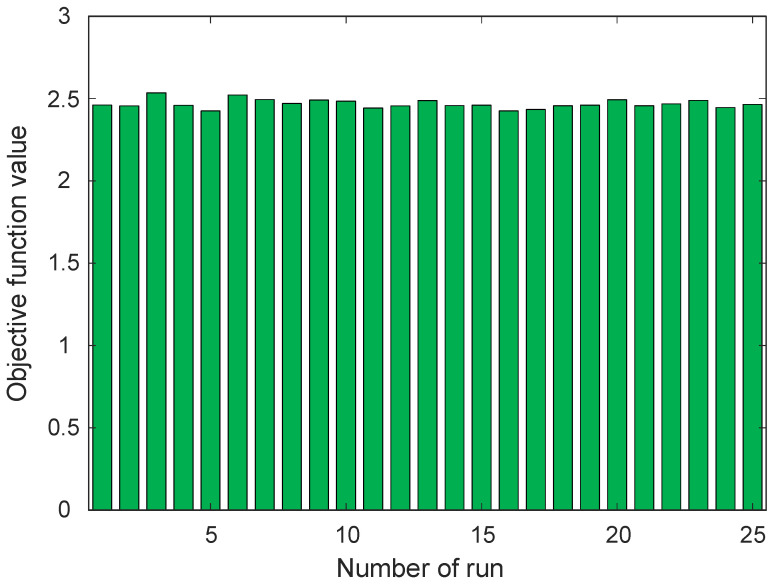
Values of objective function obtained from 25 runs.

In order to evaluate the robustness and reliability of the proposed optimization framework, the GOA was executed multiple times under identical conditions. Since metaheuristic algorithms inherently involve stochastic components, repeated runs are necessary to assess the stability of the obtained solutions and to verify whether the algorithm consistently converges toward high-quality results. The statistical indicators obtained from these independent optimization runs are summarized in Table 2, while the distribution of the objective function values across the runs is illustrated in Figure 6. As reported in Table 2, the minimum objective function value achieved by the GOA-based optimization process is 2.4251, whereas the maximum value obtained across the runs is 2.5347. The average objective value is calculated as 2.4674, which represents the typical performance level achieved by the algorithm during the optimization process. The difference between the maximum and minimum values is relatively small, indicating that the algorithm repeatedly identifies solutions of comparable quality. This observation suggests that the proposed optimization framework demonstrates stable convergence characteristics when applied to the PID tuning problem of the electric furnace temperature control system. To further evaluate the consistency of the results, the relative range defined as ∣Maximum−Minimum∣/Average×100 is also reported in Table 2. The obtained value of 4.4425% indicates that the spread of the objective function values across the optimization runs remains limited. Such a small variation reflects the ability of the algorithm to repeatedly locate solutions within a narrow performance interval, thereby demonstrating reliable search behavior. The standard deviation value of 0.0268 further confirms the stability of the optimization process. A relatively low standard deviation implies that the objective values obtained from different runs remain closely clustered around the mean value. This characteristic is particularly desirable in optimization-based controller design, since it indicates that the tuning process is not highly sensitive to the stochastic initialization of the algorithm.

The graphical representation presented in Figure 6 provides additional insight into the distribution of the objective function values obtained from the independent runs. As shown in the figure, the objective values remain concentrated within a narrow band around the average value. No extreme outliers or abrupt variations can be observed, which further demonstrates the consistent performance of the GOA during the optimization process. The relatively uniform distribution of the bars indicates that the algorithm maintains stable search behavior and avoids large fluctuations in the obtained solutions.

It should be emphasized that the small variations observed in Figure 6 are primarily attributed to the stochastic nature of the GOA. Since the optimization process is initialized with randomly generated candidate solutions in each independent run, slight differences in the obtained objective values are expected. Nevertheless, the magnitude of this variation remains limited, as evidenced by the narrow range between the minimum and maximum values and the low standard deviation reported in Table 2. The absence of extreme deviations or outliers indicates that the algorithm consistently converges toward similar high-quality solutions. Therefore, the variation observed in Figure 6 does not reflect instability; rather, it confirms the robustness and repeatability of the proposed optimization framework.

### 3.2. Evolution Curves of Objective Function and PID Controller Parameters

**Figure 7:**
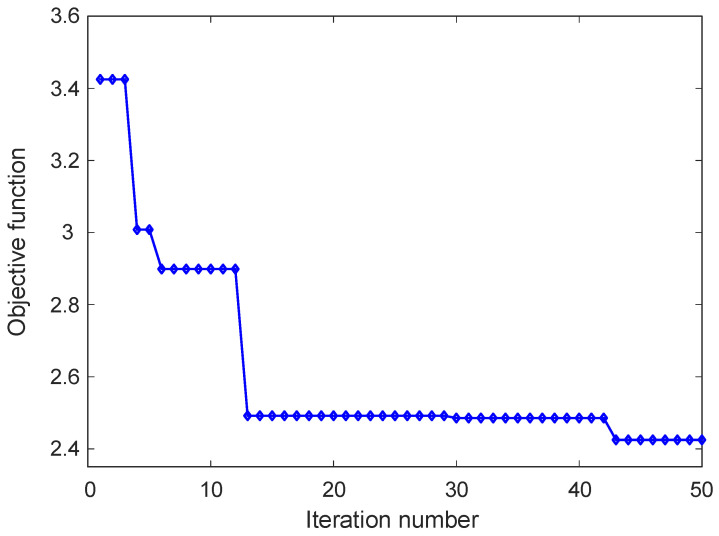
Change in objective function according to iteration number.

The convergence behavior of the GOA during the PID parameter tuning process is illustrated in Figure 7, which presents the evolution of the objective function value with respect to the iteration number. The curve provides insight into how the optimization process progressively improves the controller parameters in order to minimize the defined performance index. At the initial stage of the optimization process, the objective function value is relatively high, indicating that the initial candidate solutions do not yet provide an optimal control performance. During the first few iterations, noticeable reductions in the objective function value are observed. This rapid improvement reflects the exploratory capability of the algorithm, where different regions of the search space are investigated and inferior solutions are gradually replaced with more promising candidates. As the iteration process continues, a significant decrease in the objective function value occurs, indicating that the algorithm has identified improved controller parameter combinations. After this phase, the curve begins to stabilize and remains nearly constant over a range of iterations. This behavior suggests that the search process has entered an exploitation stage, where the algorithm focuses on refining the promising solutions that have already been identified. Toward the final iterations, a small additional improvement in the objective function value can be observed before the curve ultimately stabilizes. This gradual refinement demonstrates that the algorithm continues to explore nearby solution regions to achieve further optimization, even after the main convergence trend has been established.

**Figure 8:**
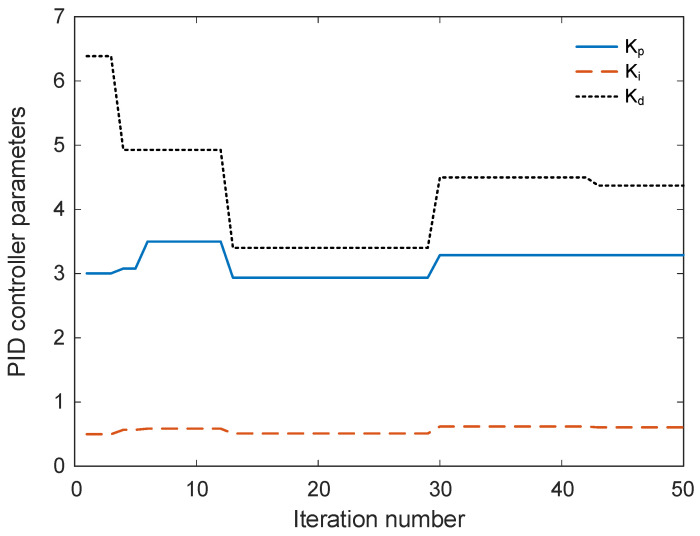
Change in controller parameters according to iteration number.

Figure 8 illustrates the evolution of the PID controller parameters $K_{p}$, $K_{i}$, and $K_{d}$ throughout the iterative optimization process carried out by the GOA. The horizontal axis represents the iteration number, while the vertical axis indicates the corresponding values of the controller parameters obtained during the search procedure. The figure therefore provides insight into how the optimization algorithm gradually adjusts the controller gains in order to improve the dynamic response of the temperature control system. At the initial stage of the optimization process, noticeable variations in the parameter values can be observed. This behavior reflects the exploratory phase of the algorithm, during which different regions of the search space are investigated in order to identify promising candidate solutions. During the early iterations, relatively larger adjustments are applied to the proportional gain $K_{p}$ and derivative gain $K_{d}$, indicating that the algorithm is actively exploring alternative controller configurations. The integral gain $K_{i}$ also exhibits moderate variation, although its changes appear less pronounced compared with the other parameters. As the optimization progresses, the magnitude of these variations gradually decreases. After approximately the middle portion of the iteration sequence, the parameter trajectories begin to stabilize. This stabilization suggests that the algorithm has identified a region of the search space containing near-optimal solutions and is therefore transitioning from exploration toward exploitation. In this phase, only minor refinements are applied to the controller gains in order to further improve the objective function value. Toward the final iterations, the values of all three parameters become nearly constant, indicating that convergence of the optimization process has been achieved. The proportional gain settles around a value slightly above three, while the integral gain stabilizes at a relatively small value, reflecting the need for gradual elimination of steady-state error without introducing excessive oscillations. Meanwhile, the derivative gain converges to a larger value compared with the other parameters, suggesting that derivative action plays a significant role in damping transient oscillations and improving system stability.

The best controller parameters identified by the GOA are presented in Table 3. These optimal parameters lead to the corresponding transfer function of the PID controller, which is fine-tuned to provide superior performance for the temperature control system. The obtained values ensure that the PID controller effectively minimizes the error, reduces overshoot, and achieves faster settling times, thereby enhancing the overall performance and stability of the temperature control system.

### 3.3. Step Response of GOA-PID Controlled System

**Figure 9:**
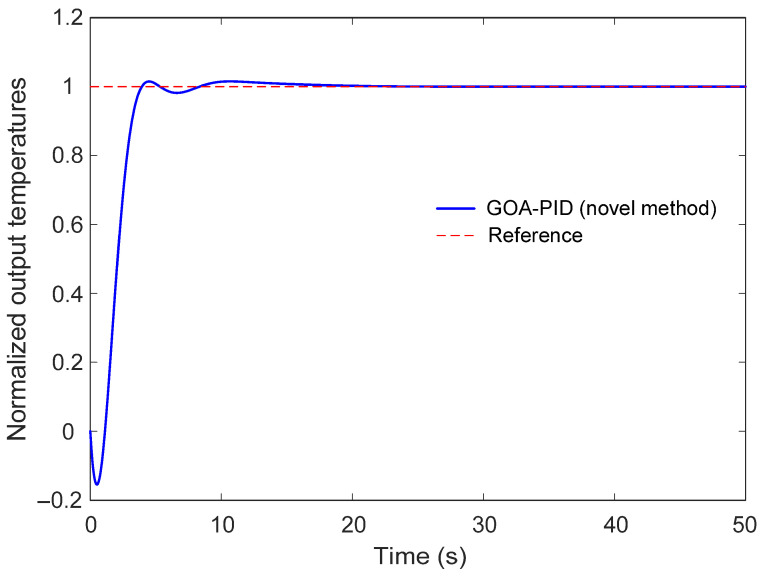
Step response of GOA-proposed PID-controlled system.

The transient behavior of the temperature control system using the PID controller tuned by the GOA is illustrated in Figure 9, while the corresponding quantitative performance indicators are summarized in Table 4. The step response analysis provides an important evaluation of how effectively the proposed control strategy regulates the furnace temperature following a change in the reference signal. As depicted in Figure 9, the closed-loop response of the GOA-PID controlled system closely follows the reference trajectory after a unit step input is applied. At the initial stage of the response, the output temperature increases rapidly toward the desired reference value, indicating that the controller provides sufficient control action to drive the system toward the target temperature. A small and well-damped overshoot can be observed during the transient phase; however, the oscillations decay quickly, and the response stabilizes smoothly around the reference level. This behavior demonstrates that the controller parameters obtained through the GOA-based optimization process achieve an appropriate balance between response speed and stability. Although derivative action is commonly associated with increased sensitivity to rapid changes in the reference signal, the results presented in Figure 9 demonstrate that such adverse effects are effectively suppressed in the proposed approach. The optimized controller exhibits only a small overshoot and quickly settles to the desired temperature level. This behavior can be attributed to the balanced tuning achieved by the GOA, which regulates the contribution of the derivative term in accordance with the overall performance objectives.

The numerical performance indices presented in Table 4 further support the observations obtained from the graphical response. The rise time of the system is 1.8509 s, indicating that the output temperature reaches the vicinity of the reference value within a relatively short period. This rapid increase reflects the capability of the optimized controller to provide prompt corrective action in response to temperature deviations. The settling time is 3.6834 s, which shows that the system stabilizes quickly after the transient phase and remains within the prescribed tolerance band around the steady-state value. Such a short settling duration is particularly desirable in thermal control applications, where prolonged transient behavior may negatively affect process efficiency.

In addition, the percentage overshoot is limited to 1.5104%, demonstrating that the proposed controller effectively suppresses excessive oscillations during the transient response. Maintaining a small overshoot is especially important for electric furnace systems, since large temperature excursions may lead to undesirable thermal fluctuations or energy inefficiencies. The low overshoot observed in the response indicates that the derivative and proportional actions of the optimized PID controller contribute to improved damping characteristics. From a practical standpoint, it is important to relate the obtained overshoot value to commonly accepted engineering limits. In thermal process control applications such as electric furnaces, overshoot is typically required to remain within a narrow range in order to prevent excessive heating and ensure product quality. In general industrial practice, overshoot values below 5% are considered acceptable, while more stringent applications often require values below 2–3%. The overshoot value of 1.5104% obtained in this study falls well within these conservative limits, indicating that the proposed GOA-based tuning approach provides a safe and reliable transient response suitable for practical temperature regulation systems.

### 3.4. Performance Comparison with Reported Methods

This section provides a performance comparison of the GOA-based approach with various reported methods for PID controller tuning, including genetic algorithm (GA) [33], Ziegler–Nichols (ZN) [34], Cohen–Coon (CC) [34], Nelder–Mead (NM) [34] and direct synthesis (DS) [34]. Table 5 displays the best obtained parameters via these approaches and presents the corresponding transfer functions.

**Figure 10:**
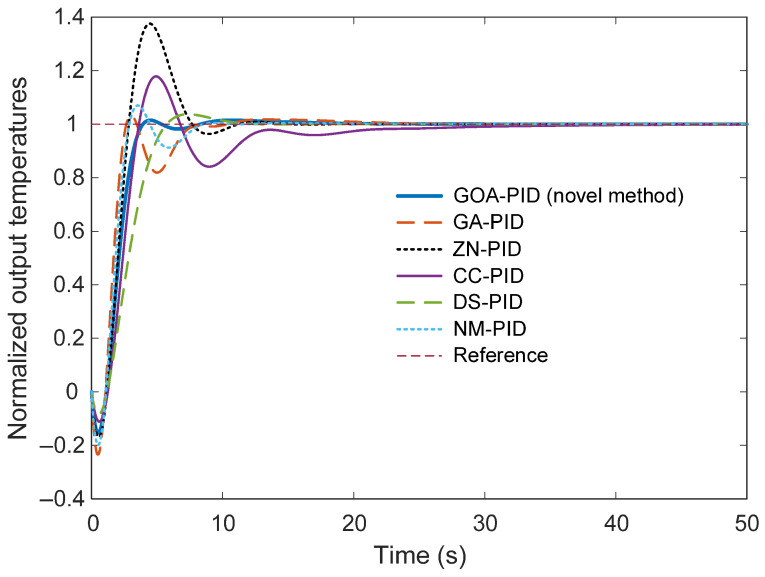
Step response of the system optimized by various approaches.

Figure 10 presents the comparative step response of the electric furnace temperature control system when the PID controller parameters are tuned using different approaches, namely the proposed GOA-PID method and several well-established techniques including GA-PID, ZN-PID, CC-PID, DS-PID, and NM-PID. The responses illustrate how each tuning strategy influences the transient behavior of the closed-loop system when subjected to a step change in the reference temperature. From the figure, it can be observed that all control strategies eventually guide the system output toward the desired reference value. However, noticeable differences appear in the transient response characteristics, particularly in terms of overshoot magnitude, oscillatory behavior, and settling duration. Among the compared approaches, the controller tuned using the proposed GOA method demonstrates a well-balanced dynamic response. The system reaches the desired temperature smoothly while maintaining a relatively small overshoot and stabilizing within a short time interval. This behavior indicates that the optimization process successfully determined a parameter set that improves both responsiveness and stability. In contrast, the classical tuning approaches exhibit less desirable transient characteristics. The Ziegler–Nichols-based controller produces the largest overshoot, where the system output significantly exceeds the reference temperature before gradually returning to the steady-state value. Such behavior may lead to undesirable thermal excursions in practical furnace operations. Similarly, the Cohen–Coon-tuned controller demonstrates pronounced oscillations and a considerably longer settling period, indicating a less stable transient response. The DS-PID and NM-PID approaches produce comparatively moderate responses, yet their settling times remain longer than that achieved by the GOA-based controller. Although the GA-PID controller achieves a relatively fast initial rise, it still exhibits noticeable oscillations before reaching steady state. These oscillatory behaviors suggest that the corresponding tuning methods do not fully balance the trade-off between response speed and stability. From a control perspective, it is also important to note that the derivative component does not induce excessive heating or instability in the proposed method. As observed in Figure 10, the GOA-based PID controller achieves a well-damped response with reduced overshoot compared to several conventional tuning approaches. This confirms that the optimization process successfully balances the proportional, integral, and derivative actions, ensuring stable operation while maintaining improved transient performance.

Table 6 summarizes the principal time-domain performance indicators obtained for the electric furnace temperature control system when different PID tuning strategies are employed. The reported metrics include rise time, settling time, and percentage overshoot, which collectively describe the transient characteristics and stability of the closed-loop response. The results indicate that the PID controller tuned using the GOA-PID achieves a well-balanced dynamic response. Although the rise time of the proposed method (1.8509 s) is not the smallest among the compared approaches, the overall transient performance is notably superior. In particular, the settling time obtained with the GOA-based tuning strategy is 3.6834 s, which is substantially shorter than those produced by the alternative methods. This rapid stabilization suggests that the optimization process successfully identified controller parameters that enable the system to quickly converge to the desired operating condition after a disturbance or reference change.

The overshoot value associated with the GOA-PID controller is also significantly lower than that of most competing approaches. With an overshoot of only 1.5104%, the proposed method demonstrates improved stability and reduced oscillatory behavior during the transient period. Such characteristics are particularly desirable in electric furnace applications, where excessive temperature excursions may negatively affect process quality and energy efficiency. By comparison, the GA-based tuning approach produces the fastest rise time (1.1378 s), indicating that the system responds more rapidly to the reference input. However, this faster response is accompanied by a noticeably longer settling time of 7.3185 s and a higher overshoot of 3.2725%. These results suggest that although the GA-PID controller reacts quickly, the transient response remains less stable and requires additional time to fully stabilize. The classical Ziegler–Nichols method exhibits the largest overshoot among the compared approaches, reaching 37.5675%. This substantial overshoot reflects the aggressive nature of the ZN tuning rule, which often prioritizes rapid response at the expense of stability. Consequently, although the rise time is relatively small, the resulting response may be unsuitable for applications requiring precise temperature regulation. Similarly, the Cohen–Coon tuning method produces a moderate rise time but suffers from a very long settling time of 21.4023 s and a relatively large overshoot of 17.8485%. These characteristics indicate a slower stabilization process and a more oscillatory transient response compared with the proposed optimization-based approach. The DS-PID and NM-PID methods demonstrate intermediate performance. While both methods provide reasonable rise times, their settling times remain significantly longer than that achieved by the GOA-PID controller. Additionally, their overshoot values are noticeably higher, indicating less effective damping of transient oscillations.

## 4. Conclusions and Potential Future Works

In this study, an optimization-based control framework was developed to improve the temperature regulation performance of an electric furnace system. A mathematical model incorporating thermal dynamics and time-delay effects was utilized as the basis for controller design. The PID controller parameters were tuned using the GOA, and a composite objective function was introduced to achieve a balanced improvement in both steady-state accuracy and transient response characteristics. The results obtained from the simulation studies demonstrate that the proposed approach provides stable and consistent optimization performance. Across 25 independent runs, the objective function values were tightly distributed, with a minimum of 2.4251, a maximum of 2.5347, and an average of 2.4674, indicating reliable convergence behavior. The optimized control system achieved a rise time of 1.8509 s, a settling time of 3.6834 s, and a low overshoot of 1.5104%. These results confirm that the proposed GOA-based PID tuning strategy effectively improves both response speed and system stability while maintaining precise temperature tracking. Comparative analyses further revealed that the proposed method provides a more balanced transient response compared with conventional tuning techniques such as GA, ZN, CC, NM, and DS methods. In particular, the proposed approach achieves significantly reduced overshoot and faster settling behavior, which are critical for preventing excessive heating and ensuring safe operation in thermal systems.

Despite these promising results, certain limitations should be acknowledged. The present study is based on simulation analysis, and experimental validation on a real electric furnace system has not yet been conducted. In addition, the investigation is limited to PID-based control structures, and more advanced control strategies such as fuzzy or fractional-order controllers have not been directly considered under identical conditions. Future research may focus on experimental implementation of the proposed method, extension to more complex and nonlinear thermal processes, and direct comparative evaluation with advanced control strategies. Furthermore, hybrid optimization frameworks and adaptive control schemes may be explored to further enhance system performance under varying operating conditions.

## Figures and Tables

**Table 1:** Parameters of GOA for optimization problem of PID-based temperature control system.

Parameter	Value
Population size	30
Maximum iteration number	50
Variable number	3
Independent run number	25
$\mathrm{Limits}\;\mathrm{of}\;K_{p}$	[1, 4]
$\mathrm{Limits}\;\mathrm{of}\;K_{i}$	[0, 2]
$\mathrm{Limits}\;\mathrm{of}\;K_{d}$	[3, 7]
s	[0, 1]
μ	[−1, 1]
S	0.88
PSRs	0.34

**Table 2:** Statistical metrics obtained for GOA-based approach.

Minimum	Maximum	Average	$\frac{\left|Maximum\;-\;Minimum\right|}{Average}\times 100$	Standard Deviation
2.4251	2.5347	2.4674	4.4425%	0.0268

**Table 3:** Best controller parameters and the corresponding transfer function obtained via GOA.

$K_{p}$	$K_{i}$	$K_{d}$	Transfer Function
3.2869	0.6056	4.3695	$\frac{-0.4916s^{3}+0.2856s^{2}+0.4249s+0.09084}{0.75s^{4}+1.333s^{3}+1.536s^{2}+0.6249s+0.09084}$

**Table 4:** Step response of proposed system.

Rise Time (s)	Settling Time (s)	Overshoot (%)
1.8509	3.6834	1.5104

**Table 5:** Obtained best controller parameters via different reported approaches and their corresponding transfer functions.

Tuning Method-Controller	$K_{p}$	$K_{i}$	$K_{d}$	Transfer Function
GA-PID	3.4066	0.6221	6.7512	$\frac{-0.7595s^{3}+0.6294s^{2}+0.441s+0.09331}{0.75s^{4}+1.065s^{3}+1.879s^{2}+0.641s+0.09331}$
ZN-PID	4.4573	1.1430	4.3455	$\frac{-0.4889s^{3}+0.1504s^{2}+0.54s+0.1714}{0.75s^{4}+1.336s^{3}+1.4s^{2}+0.74s+0.1714}$
CC-PID	3.9931	0.4144	2.6267	$\frac{-0.2955s^{3}-0.05522s^{2}+0.5523s+0.06216}{0.75s^{4}+1.529s^{3}+1.195s^{2}+0.7523s+0.06216}$
DS-PID	2.5150	0.4572	2.2864	$\frac{-0.2572s^{3}+0.06002s^{2}+0.3258s+0.06858}{0.75s^{4}+1.568s^{3}+1.31s^{2}+0.5258s+0.06858}$
NM-PID	3.7918	0.6324	5.5941	$\frac{-0.6293s^{3}+0.4125s^{2}+0.4976s+0.09486}{0.75s^{4}+1.196s^{3}+1.663s^{2}+0.6976s+0.09486}$

**Table 6:** Comparative time response metrics achieved via different reported approaches. The bold ones are the best reported values.

Tuning Method-Controller	Rise Time (s)	Settling Time (s)	Overshoot (%)
GOA-PID (novel)	1.8509	**3.6834**	**1.5104**
GA-PID	**1.1378**	7.3185	3.2725
ZN-PID	1.2840	10.1386	37.5675
CC-PID	1.7853	21.4023	17.8485
DS-PID	3.0806	9.3605	3.6937
NM-PID	1.3047	7.7546	7.0026

## Data Availability

All related data are presented within the manuscript.
